# Comparison of pericapsular nerve group block versus supra-inguinal fascia iliaca block on postoperative pain management, quadriceps muscle strength, and early functional outcomes after arthroscopic treatment of femoroacetabular impingement: a prospective, randomized clinical study

**DOI:** 10.1093/jhps/hnaf039

**Published:** 2025-08-04

**Authors:** Mohamed Abd El-Radi, Hatem G Said, Essam S Abdallah, Mahmoud M Kamel, Saeid M Elsawy, Ayman F AbdelKawi, Mohamed Abd El-Tawab, Safaa S Noaman

**Affiliations:** Department of Orthopedic and Trauma Surgery, Faculty of Medicine, Assiut University, University Road, Assiut 71515, Egypt; Department of Orthopedic and Trauma Surgery, Faculty of Medicine, Assiut University, University Road, Assiut 71515, Egypt; Department of Anesthesiology, Intensive Care, and Pain Management, Faculty of Medicine, Assiut University, University Road, Assiut 71515, Egypt; Department of Anesthesiology, Intensive Care, and Pain Management, Faculty of Medicine, Assiut University, University Road, Assiut 71515, Egypt; Department of Anesthesiology, Intensive Care, and Pain Management, Faculty of Medicine, Assiut University, University Road, Assiut 71515, Egypt; Department of Orthopedic and Trauma Surgery, Faculty of Medicine, Assiut University, University Road, Assiut 71515, Egypt; Department of Orthopedic and Trauma Surgery, Faculty of Medicine, Assiut University, University Road, Assiut 71515, Egypt; Department of Anesthesiology, Intensive Care, and Pain Management, Faculty of Medicine, Assiut University, University Road, Assiut 71515, Egypt

## Abstract

This study compared the effects of pericapsular nerve group (PENG) block and supra-inguinal fascia iliaca compartment block (FICB) on quadriceps strength, postoperative analgesia, and functional scores following hip arthroscopy (HA) for patient complaining of femoroacetabular impingement (FAI) under general anaesthesia. A prospective, randomized, and controlled trial recruited a total of 48 patients who had either PENG group or supra-inguinal FICB as a pain management modality. The primary outcomes were the postoperative quadriceps muscle power, and the secondary outcomes included pain scores, opioid consumption, and functional scores; the modified Harris hip score (mHHS) and international hip outcome tool-12 (iHOT-12) score. All patients in PENG group were capable of performing a straight leg raise with hip flexion of >15°. In contrast, two patients in FICB group had postoperative falls and six patients (25%) were unable to perform a straight leg raise at 24 h postoperatively. At the time of admission to the post-anaesthesia care unit and at 6, 12, and 24 h postoperatively, peak visual analogue scale (VAS) scores at rest were 6, 5, 4, and 4, respectively, in PENG group and 8, 7, 6, and 5, respectively, in FICB group (*P* < .001). Total opioid consumption in the 24 h postoperatively was lower in the PENG group compared to the FICB group (mean, 18.5 ± 9.4 versus 26.8 ± 9.6; *P* < .05). Significant improvement was observed (6 and 12 months postoperatively) regarding iHOT-12 score in the PENG group (70 ± 24.33, 82.85 ± 11.45) compared to the FICB group (59.04 ± 22.66, 64.1 ± 17.42) (*P* = .02, *P* < .001, respectively).

## INTRODUCTION

Adequate pain management after hip arthroscopy (HA) is essential for early weight bearing and patient satisfaction [1]. However, due to the complexity of hip joint innervation, the efficient modality of regional analgesia remains controversial [2].

The anterior capsule of the hip joint is innervated by the femoral, accessory obturator, and obturator nerves [3]. To block the articular branches from these three nerves, iliopubic eminence and inferomedial acetabulum were proposed as crucial bony landmarks by several anatomical studies [4].

The pericapsular nerve group (PENG) block has been documented to have an efficient analgesic effect, with reduced pain scores and absence of quadriceps muscle weakness in patients with hip fracture and those who had undergone total hip arthroplasty (THA) [5, 6]. Desmet et al. demonstrated that the supra-inguinal fascia iliaca compartment block (FICB) led to reduced morphine consumption and lower pain scores after THA [7]. However, the supra-inguinal FICB carries a potential risk of quadriceps weakness, which could hinder immediate ambulation.

Femoroacetabular impingement (FAI) is a condition caused by repeated abnormal loading of the hip joint, which leads to unusual contact between the bones, the acetabular labrum, and the cartilage, potentially causing labral tears and cartilage damage. FAI is classified into three types: cam type, which involves bone overgrowth at the junction of the femoral head and neck; pincer type, caused by excessive bone growth on the acetabular rim; and mixed type, which has features of both cam and pincer deformities. HA is commonly used to treat FAI by removing the excess bone on both the femoral and acetabular sides, allowing for labral repair and the treatment of cartilage injury [8–10].

In this study, we hypothesized that the PENG block would preserve quadriceps muscle power and result in less opioid consumption compared to the supra-inguinal FICB following HA. Our primary outcomes were the postoperative quadriceps muscle power, and the secondary outcome measures included pain scores, opioid consumption, and functional scores; the modified Harris hip score (mHHS) and international hip outcome tool-12 (iHOT-12) score.

## MATERIALS AND METHODS

### Study population

This prospective, double-blinded, randomized clinical study protocol was approved by the Institutional Review Board and Research Ethics Committee under number (#17101178), on 27 August 2020, and registered at ClinicalTrials.gov (NCT0443419). The study adhered to the Consolidated Standards of Reporting Trials (CONSORT) statement and the declaration of Helsinki. Written informed consent was obtained from all the trial participants. Patients aged 18 years or older with an American Society of Anesthesiologists physical status I or II, who were scheduled for elective, unilateral primary HA for FAI and labral tear under general anaesthesia, were enrolled in this study between September 2020 and September 2022. Exclusion criteria were allergy or intolerance to any of the drugs used in the study, pre-existing neurologic or anatomic deficits in the lower extremities, cognitive disorders that may impair pain evaluation, and inflammatory joint diseases.

### Study design

Enrolled patients were randomly assigned to receive either the PENG block (PENG group) or the supra-inguinal FICB (FICB group) on the day of the surgery. An independent investigator, who was not involved in patient care or preoperative assessment, used sealed envelopes to randomly assign patients to either the PENG block or the FICB at a 1:1 ratio. The envelopes were given to the anaesthesiologist performing the procedures, who was not part of the study. This ensured that the surgeons, investigators, and patients were unaware of the group assignments during the study period.

### Interventions

**Figure 1:**
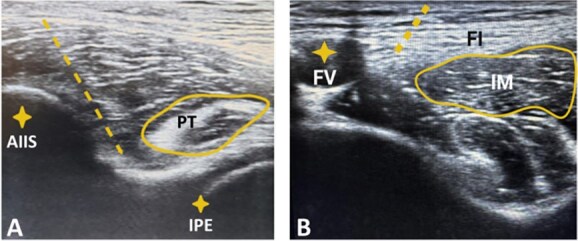
(A) Ultrasound image demonstrating the PENG block; anterior inferior iliac spine (AIIS), psoas tendon (PT), iliopectineal eminence (IPE), and dotted arrow represents needle direction. (B) Ultrasound image demonstrating the FICB; iliacus muscle (IM), fascia iliaca (FI), femoral vessels (FV), and dotted line represents needle direction.

All patients were sedated with intravenous (IV) midazolam (0–4 mg) and local anaesthesia with lidocaine 2% before induction of the blockade. An ultrasound scanner was used in all cases (GE Logiq F6, GE Healthcare, Chicago, IL) for PENG block and FICB. Both blocks were performed in the supine position by an anaesthetist who had efficient experience in regional block around the hip joint. PENG block (Fig. 1A) was performed according to the guidance of Girón-Arango et al. [5] with 20 ml of 0.5% bupivacaine injected between the iliopsoas tendon anteriorly and pubic ramus posteriorly. FICB was done according to the method of Hebbard et al. [11] through the injection of 20 ml of 0.5% bupivacaine into the supra-inguinal fascia iliaca compartment (Fig. 1B).

**Figure 2:**
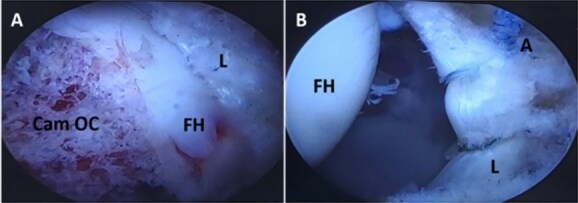
Left hip joint arthroscopic images. (A) Peripheral compartment assessment indicates femoral head (FH), labrum (L), and cam osteochondroplasty (Cam OC). (B) Central compartment assessment after traction indicates femoral head (FH), labrum (L), and anchor placement (A).

All surgical procedures were performed under general anaesthesia. After induction with 100 mcg IV fentanyl and propofol (2–2.5 mg/kg), and placement of the endotracheal tube, balanced anaesthesia was maintained with sevoflurane. IV fentanyl was administered as required by the anaesthesiologist who was blinded to the group designation of the patient. All patients received 4 mg of IV dexamethasone before skin incision and 4 mg of IV ondansetron at the end of the procedure for postoperative nausea and vomiting prophylaxis. All included patients had the diagnosis of FAI confirmed by clinical examination, plain radiograph (measuring alpha angle), and magnetic resonance arthrogram (MRA) to ensure the site of labral injury. All patients underwent standard arthroscopic protocol for FAI through osteochondroplasty and labral repair using all-suture anchors (Fig. 2). Postsurgical pain was assessed repeatedly by an investigator blinded to the group designation of the patients and was treated with IV nalbuphine as required to achieve a VAS score of 4 or less.

### Outcome assessments

Measurements of quadriceps strength were assessed by an investigator blinded to the group designation of the patient on both the surgical and nonsurgical leg to evaluate the effects of the regional technique on leg strength using the straight leg raising test at >15° hip flexion.

We assessed the level of pain at rest and during movement (45-degree active hip flexion with the knee flexed at 45°) before the surgery, and at 6, 12 (only at rest), and 24 h after the surgery in the post-anaesthesia care unit (PACU). We used an 11-point numeric rating scale (NRS: 0 = no pain, 10 = worst imaginable pain) to measure the intensity of pain. Cumulative opioid consumption at 24 h following surgery was recorded. Functional scores including mHHS and iHOT-12 score were addressed for each patient enrolled in the study preoperatively, and at 6 months and 1 year postoperatively. All outcomes and perioperative data were collected by an investigator blinded to the group allocation.

### Statistics

To obtain a power of .80 (1 − *β*) with an *α* of .05, the calculated sample size was 23 patients per group. Based on data normality, continuous variables were analysed using the independent *t*-test or Mann–Whitney *U* test. Categorical variables were analysed utilizing the chi-squared test or Fisher’s exact test. Values are presented as mean ± standard deviation, median (interquartile range), or the number of patients (proportion). The balance of patient and operation characteristics between the randomized groups was analysed by calculating the standardized difference, defined as the difference in proportions or means divided by the pooled standard deviation. All analyses were performed using SPSS version 23.0 software (IBM Corporation, Armonk, NY). *P*-value <.05 was considered statistically significant.

## RESULTS

### Patient and operation characteristics

**Figure 3:**
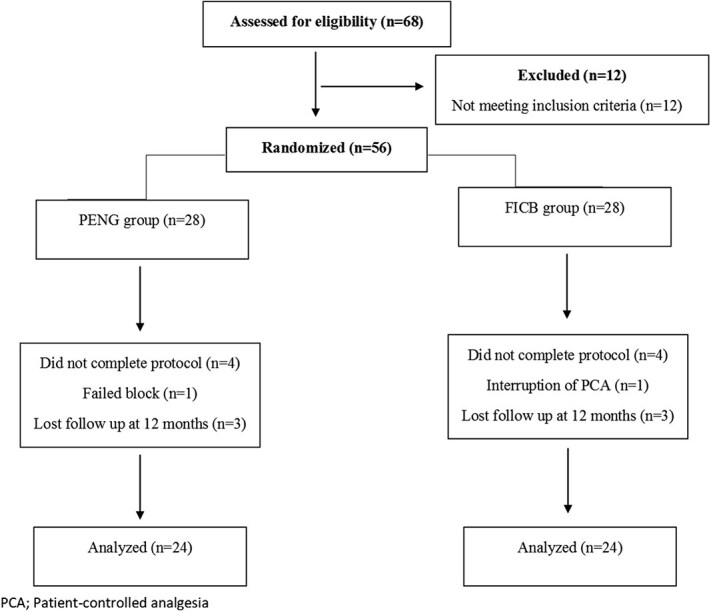
Study flowchart.

Of the 68 patients assessed for eligibility, 12 were excluded, leaving 56 patients who were enrolled. Eight patients were excluded from analysis due to lost follow up at 12 months (*n* = 6; 3 in each group), a failed block (*n* = 1, in the PENG group), and interruption of patient-controlled analgesia (PCA) (*n* = 1, in the FICB group). Consequently, 48 patients were included in the final analysis (Fig. 3). Patient baseline characteristics are detailed in Table 1. Clinical, radiological, and operative data are shown in Table 2.

**Table 1:** Patients baseline characteristics.

**Items**	**PENG group (*n* = 24)**		**FICB group (*n* = 24)**	
	**No.**	**%**	**No.**	**%**
Age				
<30 years	4	16.7	2	8.3
30–40 years	16	66.7	20	83.3
>40 years	4	16.7	2	8.3
Sex				
Male	19	79.2	18	75.0
Female	5	20.8	6	25.0
Body mass index				
Normal weight (18.5–24.9 kg/m^2^)	18	75.0	16	66.7
Overweight (25–29.9 kg/m^2^)	5	20.8	8	33.3
Obese (30 or higher kg/m^2^)	1	4.2	0	0.0

**Table 2:** Clinical, radiological, and operative data.

**Items**	**PENG group (*n* = 24)**		**FICB group (*n* = 24)**		**PENG group (*n* = 24)**	**FICB group (*n* = 24)**
	**No.**	**%**	**No.**	**%**	**Mean ± SD**	**Mean ± SD**
Type of femoroacetabular impingement (FAI)						
Cam	6	25.0	6	25.0	**–**	**–**
Pincer	0	0.0	3	12.5	**–**	**–**
Mixed (cam and pincer)	18	75.0	15	62.5	**–**	**–**
Side						
Right	16	66.7	14	58.3	**–**	**–**
Left	8	33.3	10	41.7	**–**	**–**
Duration of symptoms in months	**–**	**–**	**–**	**–**	22.5 ± 19.36	16 ± 0
Flexion-abduction-external rotation test						
Positive	19	79.2	14	58.3	**–**	**–**
Negative	5	20.8	10	41.7	**–**	**–**
Flexion, adduction, and internal rotation test						
Positive	23	95.8	24	100.0	**–**	**–**
Negative	1	4.2	**–**	**–**	**–**	**–**
Labral tear in MRA	24	100.0	24	100.0	**–**	**–**
Preoperative alpha angle	**–**	**–**	**–**	**–**	63.18 ± 9.06	61.58 ± 6.19
Postoperative alpha angle	**–**	**–**	**–**	**–**	45.58 ± 4.97	53 ± 2.32
Tönnis classification of arthritic changes	**–**	**–**	**–**	**–**	1.17 ± 2.98	2.31 ± 4.06
Treatment of labrum (labral repair using anchors suturing)	24	100.0	24	100.0	**–**	**–**
Number of anchors						
Two	17	70.8	22	91.7	**–**	**–**
Three	6	25.0	2	8.3	**–**	**–**
Four	1	4.2	**–**	**–**	**–**	**–**

MRA; Magnetic resonance arthrography

### Quadriceps strength

No patients in the PENG group experienced clinically significant quadriceps weakness, complications, or postoperative falls. All patients were capable of performing a straight leg raise with hip flexion of >15°. In contrast, two patients in the FICB group had postoperative falls and six patients (25%) were unable to perform a straight leg raise at 24 h postoperatively. However, they completely recovered at the first clinic follow-up, 2 weeks postoperative. This difference between the two groups was clinically significant, with a *P*-value <.001.

### Pain outcomes

Pre-block pain scores were similar between both groups (*P* = .782). A significant difference in dynamic pain scores (active hip flexion up to 45°) was observed between both groups at 24 h postoperatively. Median pain scores were 3 in the PENG group and 5.5 in the FICB group, with a maximum score of 5 in PENG group and 9 in FICB group (*P* < .001). At the time of admission to the PACU and at 6, 12, and 24 h postoperatively, median pain scores in the PENG group were 3, 2, 2, and 2, respectively, and in the FICB group were 6, 5, 4, and 3, respectively. Peak VAS scores at rest were 6, 5, 4, and 4, respectively, in PENG group and 8, 7, 6, and 5, respectively, in FICB group (*P* < .001).

### Postoperative opioid consumption and analgesia reequipment

No significant difference in intraoperative opioid administration was observed between both groups, with all patients receiving 100 mcg of fentanyl during induction of anaesthesia. Patients who had PENG block significantly had lower postoperative opioid use compared to the FICB group. Total opioid consumption in the 24 h postoperatively was lower in the PENG group compared to the FICB group (mean, 18.5 ± 9.4 versus 26.8 ± 9.6; *P* < .05). The total dose of analgesia in 24 h postoperatively and the first analgesia demand are shown in Table 3.

**Table 3:** Total dose of analgesia in 24 h postoperatively and first analgesia demand.

	**Block**	**Mean**	**SD**	**Significance**
Total dose of analgesia in mg	PENG	9.8	8.7	.001
	FICB	21.5	11.7	
Frist analgesic demand in hours	PENG	9.3	7.9	.001
	FICB	3.6	1.6	

PENG, pericapsular nerve group; FICB, fascia iliaca compartment block; SD, standard deviation

### Functional clinical scores

**Figure 4:**
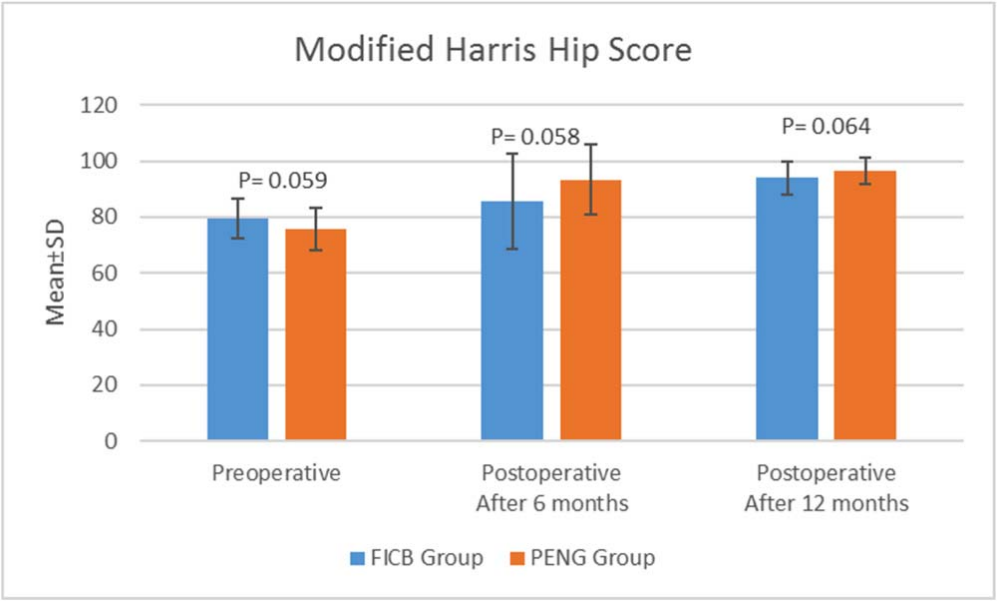
Modified Harris hip score of the studied patients.

There was no statistically significant difference (*P* = .059) between the PENG group (73.63 ± 6.43) and the FICB group (77.53 ± 7.26) regarding preoperative mHHS. Improvement was observed in mHHS scores at 6- and 12-months post-rehabilitation in both the PENG group (93.26 ± 10.41, 97.63 ± 4.81) and FICB group (83.5 ± 19.04, 91.95 ± 6.78). However, the difference was not statistically significant (*P* = .05, *P* = .06, respectively) (Fig. 4).

**Figure 5:**
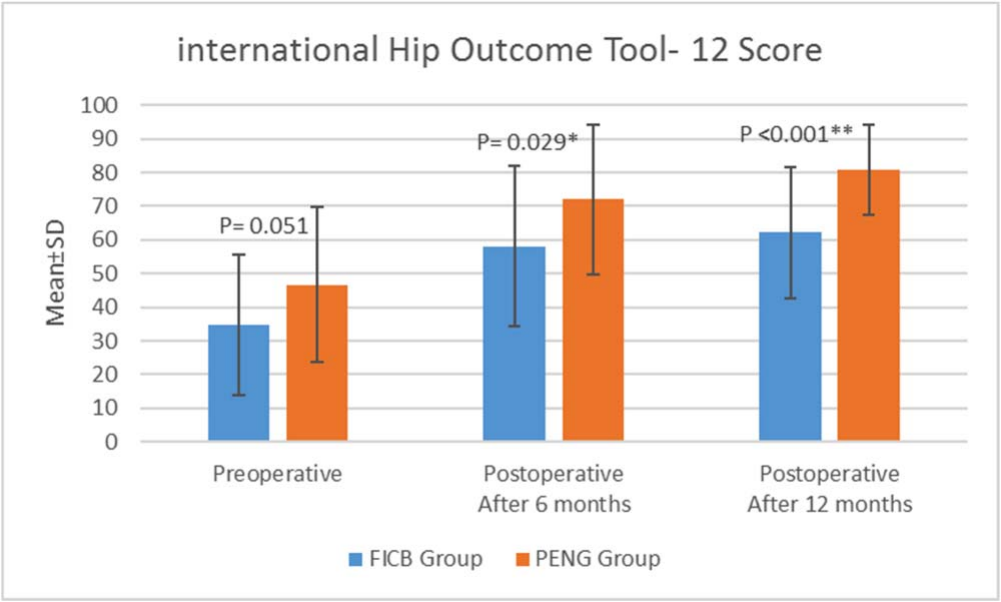
International hip outcome tool-12 score of both groups.

Furthermore, no statistically significant difference (*P* = .051) between the PENG group (44.7 ± 25.13) and the FICB group (36.83 ± 18.94) regarding preoperative iHOT-12 score. A statistically significant improvement was observed (6 and 12 months postoperatively after rehabilitation) regarding iHOT-12 score in the PENG group (70 ± 24.33, 82.85 ± 11.45) compared to the FICB group (59.04 ± 22.66, 64.1 ± 17.42) (*P* = .02, *P* < .001, respectively) (Fig. 5).

## DISCUSSION

In this study, we found a significant clinical difference as regard quadriceps muscle strength in the operated limb in the PENG group compared to the FICB group. Moreover, we found a significant difference in postoperative pain scores and opioid consumption up to postoperative 24 h between patients undergoing HA for FAI treatment who received the PENG block and the supra-inguinal FICB. Postoperative iHOT-12 scores significantly improved in the PENG group compared to the FICB at 6 months and 12 months follow up.

The PENG block is a technique used to manage pain by selectively blocking the articular branches that supply the anterior hip joint capsule while preserving the main nerves of the lumbar plexus. This approach helps to maintain muscle strength and motor function [5]. Lin et al. demonstrated that patients undergoing hip fracture surgery who received a PENG block experienced less postoperative pain compared to those who received a femoral nerve block (FNB) [12].

On the other hand, Aliste et al. documented that between patients receiving the PENG block and the supra-inguinal FICB, no differences were found in static and dynamic pain scores during postoperative 48 h, as well as cumulative opioid consumption at 24 and 48 h after THA under spinal anaesthesia [13].

Due to the rising number of HA procedures, there is a growing need to establish standardized postoperative pain management protocols for patients undergoing FAI surgeries. Various studies [1, 14, 15] have examined different approaches to anaesthesia and analgesia during HA, but there is currently no clear consensus on the optimal method of anaesthesia. Analgesia modalities such as nerve blocks, intraoperative intraarticular or extracapsular anaesthetic injections, opioids, and nonsteroidal anti-inflammatory drugs are commonly used, but the increased use of postoperative opioids is associated with higher rates of opioid-related side effects and delayed hospital discharge [1, 15, 16].

As the use of peripheral nerve block in HA is a growing approach, there is a lack of evidence-based data, and further studies of multiple alternative regional anaesthetic procedures, such as lumbar plexus blocks, FNBs, lumbar paravertebral blocks, and FICB, are required [5, 17–19].

Regional anaesthesia plays a significant role in postoperative pain management although some of these common techniques have limitations and complications. FNB has been shown to significantly reduce pain, but has also been associated with an increased incidence of postoperative falls [20, 21].

Along with previous studies, the present study showed that the PENG block group had a preserved quadriceps power at 24 h postoperatively with the ability to perform straight leg raises without postoperative falls, compared to the FICB group following HA. Aliste et al. reported that the PENG block resulted in a lower incidence of quadriceps motor power block at 3 and 6 h after THA, compared with the supra-inguinal FICB [13]. Moreover, the quadriceps strength was not quantitatively measured using a dynamometer. The PENG block in this study did not encounter any serious difficulty with needle placement, despite the possibility of unintended motor blockade resulting from the use of medial local anaesthetic injection targeting specific anatomical landmarks [22, 23]. Aliste et al. performed supra-inguinal FICB with 40 ml levobupivacaine 0.25% [13]. The studies by Desmet et al. and Gasanova et al. reporting the higher rate of quadriceps motor block following supra-inguinal FICB used 40 ml of ropivacaine 0.5% and 60 ml of ropivacaine 0.5% as the injectate, respectively [7, 24].

PENG block dramatically decreased median pain scores at rest and on movement at all measured time points through the postoperative period. Furthermore, the PENG group had less total opioid consumption postoperatively. First analgesic demand (i.e. time before requiring first opioid dose) was significantly increased in the PENG group compared to the FICB group (400 versus 180 min, *P* = .002).

PENG block might be considered a better postoperative analgesic option than peripheral nerve blocks, such as FNB, FICB, or lumbar plexus block in HA [25, 26]. However, PENG block has several limitations, including the need for deep instillation and poor vision of the needle tip [27]. To avoid complications, such as quadriceps weakness, the medication must be instilled deeply into the psoas tendon [22].

Moreover, Behrends et al. [28] reported that routine use of the FICB is not recommended as it caused weakness in the quadriceps muscles rather than providing analgesia effect after arthroscopic surgeries of the hip joint.

Both groups demonstrated improvement in iHOT-12 scores at 6 and 12 months postoperatively. However, the PENG group showed a more significant improvement. The substantial enhancement in iHOT-12 score in the PENG group may be attributed to preserved quadriceps muscle power, which influences early stages of rehabilitation such as squats, stair climbing, and the ability to resume regular exercise regimens.

To the best of our knowledge, this is the first prospective randomized study that evaluated the effect of PENG block versus FICB on FAI patients who underwent hip arthroscopic osteochondroplasty and labral repair. Furthermore, the selection of single hip pathology made a point of strength that unifies the patient stratification.

However, to validate the efficacy of the PENG block following HA, a larger sample size and a longer follow-up duration are essential. Further studies are necessary to identify ideal clinical outcomes and evaluate the applicability of the PENG block to a wider range of indications in HA.

## CONCLUSION

PENG block is easily performed in the preoperative setting, and appears to spare motor function while providing a prolonged sensory pain relief and improving hip functional assessment scores after HA in comparison to other alternative conventional regional nerve blocks such as FICB.

## Data Availability

The datasets used in the current study are available from the corresponding author upon reasonable request.
